# Multiple antimicrobial resistance and outcomes among hospitalized patients with complicated urinary tract infections in the US, 2013–2018: a retrospective cohort study

**DOI:** 10.1186/s12879-021-05842-0

**Published:** 2021-02-08

**Authors:** Marya D. Zilberberg, Brian H. Nathanson, Kate Sulham, Andrew F. Shorr

**Affiliations:** 1 EviMed Research Group, LLC, PO Box 303, Goshen, MA 01032 USA; 2 OptiStatim, LLC, 25 Willow Circle, Longmeadow, MA 01106 USA; 3 Spero Therapeutics, 675 Massachusetts Avenue, Cambridge, MA 02139 USA; 4 Washington Hospital Center, 110 Irving Street NW, Washington, DC 20010 USA

**Keywords:** Complicated UTI, Antimicrobial resistance, Epidemiology, Microbiology, Outcomes, Hospitalization

## Abstract

**Background:**

Complicated urinary tract infection (cUTI) is common among hospitalized patients. Though carbapenems are an effective treatment in the face of rising resistance, overuse drives carbapenem resistance (CR). We hypothesized that resistance to routinely used antimicrobials is common, and, despite frequent use of carbapenems, associated with an increased risk of inappropriate empiric treatment (IET), which in turn worsens clinical outcomes.

**Methods:**

We conducted a retrospective cohort study of patients hospitalized with a culture-positive non-CR cUTI. Triple resistance (TR) was defined as resistance to > 3 of the following: 3rd generation cephalosporins, fluoroquinolones, trimethoprim-sulfamethoxazole, fosfomycin, and nitrofurantoin. Multivariable models quantified the impact of TR and inappropriate empiric therapy (IET) on mortality, hospital LOS, and costs.

**Results:**

Among 23,331 patients with cUTI, 3040 (13.0%) had a TR pathogen. Compared to patients with non-TR, those with TR were more likely male (57.6% vs. 47.7%, *p* < 0.001), black (17.9% vs. 13.6%, p < 0.001), and in the South (46.3% vs. 41.5%, *p* < 0.001). Patients with TR had higher chronic (median [IQR] Charlson score 3 [2, 4] vs. 2 [1, 4], *p* < 0.001) and acute (mechanical ventilation 7.0% vs. 5.0%, p < 0.001; ICU admission 22.3% vs. 18.6%, *p* < 0.001) disease burden. Despite greater prevalence of empiric carbapenem exposure (43.3% vs. 16.2%, *p* < 0.001), patient with TR were also more likely to receive IET (19.6% vs. 5.4%, p < 0.001) than those with non-TR. Although mortality was similar between groups, TR added 0.38 (95% CI 0.18, 0.49) days to LOS, and $754 (95% CI $406, $1103) to hospital costs. Both TR and IET impacted the outcomes among cUTI patients whose UTI was not catheter-associated (CAUTI), but had no effect on outcomes in CAUTI.

**Conclusions:**

TR occurs in 1 in 8 patients hospitalized with cUTI. It is associated with an increase in the risk of IET exposure, as well as a modest attributable prolongation of LOS and increase in total costs, particularly in the setting of non-CAUTI.

**Supplementary Information:**

The online version contains supplementary material available at 10.1186/s12879-021-05842-0.

## Background

Complicated urinary tract infections (cUTI) are a leading infection-related reason for acute hospitalization. A cUTI can also arise as a nosocomial complication that represents a key focus for prevention among hospitalized patients. Frequently occurring in the presence of an indwelling catheter, catheter-associated urinary tract infections (CAUTIs) are the publicly reportable subset of cUTI, and are included in the Hospital Compare metrics by the United States Centers for Medicare and Medicaid Services [1, 2]. Irrespective of whether community- (CO) or hospital-onset (HO), cUTIs place a significant burden on the healthcare system.

Rising rates of antimicrobial resistance to commonly used antibiotics are adding hurdles to patient care [3]. Particularly troubling is the growing prevalence of extended-spectrum beta-lactamase producing Enterobacteraciae (ESBLs) at the time that the in vitro susceptibility rates to fluroquinolones and other routinely employed antimicrobials for cUTI, such as 3rd generation cephalosporins and trimethoprim-sulfamethoxazole, are diminishing [4–7]. These shifts are making it difficult for clinicians to target empiric therapy.

In order to weigh the risks of therapeutic failure when administering standard treatment regimens, we examined the prevalence of overlapping resistance to commonly utilized antibiotics in cUTI and its impact on the outcomes. We also estimated the rate of inappropriate empiric therapy (IET) as a function of compound resistance in cUTI, and of its impact on morbidity and mortality.

## Methods

### Ethics statement

Because this study used already existing fully de-identified data, it was exempt from ethics review under US 45 CFR 46.101(b)4 [8, 9]. Current analyses were performed within the same cohort of patients as those reported in citation 10.

### Study design and patient population

We conducted a multi-center retrospective cohort study of hospitalized patients with culture-positive carbapenem-susceptible cUTI to explore the prevalence and impact of resistance to commonly used non-carbapenem empiric regimens. The case identification approach relied on a previously published algorithm [10].

The cohort was followed longitudinally until discharge or death in the hospital. Survivors were followed for additional 30 days for the outcome of 30-day readmission.

### Data source

The data for the study derived from Premier Research database, an electronic laboratory, pharmacy and billing data repository, for years 2013 through 2018. The database has been described in detail previously [9–13]. We used data from a subset of approximately 180 US institutions who submitted microbiology data during the study time frame.

### Baseline measures and cUTI classification

cUTI was classified as CO if present on admission (POA) or if index culture was drawn within first 2 hospital days. CO cUTI was further classified as healthcare-associated (HCA) if one or more of the following risk factors was present: 1). Hospitalization within prior 90 days, 2). Hemodialysis, 3). Admission from a long-term care facility, 4). Immune suppression [10]. All other CO infections were community-acquired (CA). All cUTIs occurring on or after hospital day 3 were considered HO.

In addition to infection classification, patient factors examined included history of exposure to antibiotics within prior 90 days; antibiotics exposure during the index hospitalization prior to cUTI onset if HO; previous Emergency Department visit with a UTI within 90 days of the index culture; hospital structural characteristics (size, teaching status, urbanicity, census region); demographic variables, and comorbid conditions. Charlson comorbidity score was computed as a measure of the burden of chronic illness.

### Infection and treatment variables

ICU admission, mechanical ventilation, presence of severe sepsis or septic shock, dialysis, and vasopressor use were used as markers for acute disease severity. Organisms and their susceptibilities were identified, and empiric antibiotic treatment was considered appropriate if the patient received coverage that included the corresponding organism within two days of the culture being obtained. All other coverage was considered IET.

### Microbiology and susceptibilities

Organisms were classified as susceptible (S), intermediate (I), or resistant (R). For the purposes of the current analyses, I and R were grouped together as resistant. We determined each isolate’s susceptibility status to each of the common antimicrobials of interest (3rd generation cephalosporins, [C3]; fluoroquinolones [FQ]; trimethoprim-sulfamethoxazole [TMP-SMZ]; fosfomycin [FFM]; and nitrofurantoin [NTF]). Triple-drug resistance (TR) was defined as resistance to at least three separate antimicrobials or classes of interest (i.e., resistance to at least one of the member drugs within the class).

First detection of an organism served as the index culture. To be considered culture-positive, the patient had to grow out a qualifying common bacterium in urine or blood. Organisms of interest included Enterobacteriaceae, *P. aeruginosa, A. baumannii, E. faecium, E. faecalis* [9].

### Outcome variables

Hospital mortality served as the primary outcome, and 30-day readmission, hospital length of stay (LOS) and costs as secondary outcomes. Exploratory outcomes were incidence of *C. difficile* infection (CDI), cUTI recurrence (defined as a new positive culture following a >/=3-day hiatus in antimicrobial administration), and development of TR. All antimicrobial susceptibility testing was done at the individual hospital in accordance with its standards and consistent with in vitro break-points in place at the time.

### Statistical analyses

We report standard descriptive statistics to compare TR and non-TR groups across all demographics, comorbidities, infection characteristics, hospital characteristics and processes, and hospital outcomes. Continuous variables are reported as means with standard deviations and as medians with interquartile ranges (IQR). Differences between mean values were tested via Student’s t-test, and between medians using the Mann-Whitney U test. Categorical data are summarized as proportions, with the Chi-square or Fisher’s exact test used to examine inter-group differences. Statistical significance was set at *p* < 0.05. Because of the large sample size, statistical significance may not equate to clinical significance.

We developed multilevel mixed-effects regression models to examine the impact of TR on hospital outcomes with hospitals treated as random effects. For continuous variables (LOS and costs), we used generalized linear models with a gamma distribution, and applied a logarithmic link function. For categorical variables (mortality, 30-day readmission, recurrence, incident TR and CDI), we used logistic regression. We bracketed the point estimates with 95% confidence intervals (CI).

## Results

Among 23,331 patients with cUTI who met the enrollment criteria 28,192 organisms were isolated [9]. The pathogens, TR prevalence over the study timeframe, and resistance rates to individual antimicrobials are in Table 1, Supplemental Table 1, as well as in the Supplemental Table 4 in citation 10. The prevalence of TR in the cohort was 13.0% (*n* = 3040, Table 2). Compared to patients with non-TR, those with TR were more likely male (57.6% vs. 47.7%, *p* < 0.001), black (17.9% vs. 13.6%, *p* < 0.001), and in the South (46.3% vs. 41.5%, p < 0.001), and had a higher chronic disease burden (median [IQR] Charlson score 3 [2, 4] vs. 2 [1, 4], p < 0.001). Patients with TR were hospitalized at centers with higher median prevalence of both C3R and TR (Table 2).

**Table 1:** Microbiology of cUTI

	TR	%	non-TR	%	All	%^a^
	*N* = 3040		***N*** = 20,291		***N*** = 23,331	
**Organism**						
*Escherichia coli*	1470	48.36%	10,040	49.48%	11,510	49.33%
*Klebsiella pneumoniae*	530	17.43%	2865	14.12%	3395	14.55%
*Pseudomonas aeruginosa*	244	8.03%	2836	13.98%	3080	13.20%
*Proteus mirabilis*	916	30.13%	1823	8.98%	2739	11.74%
*Enterococcus faecalis*	195	6.41%	2251	11.09%	2446	10.48%
*Enterococcus spp.*	80	2.63%	856	4.22%	936	4.01%
*Enterobacter cloacae*	118	3.88%	547	2.70%	665	2.85%
*Klebsiella oxytoca*	32	1.05%	469	2.31%	501	2.15%
*Providencia spp*	133	4.38%	340	1.68%	473	2.03%
*Citrobacter freundii*	40	1.32%	393	1.94%	433	1.86%
*Morganella morganii*	173	5.69%	229	1.13%	402	1.72%
*Serratia marcescens*	39	1.28%	321	1.58%	360	1.54%
*Enterococcus faecium*	27	0.89%	312	1.54%	339	1.45%
*Enterobacter aerogenes*	8	0.26%	276	1.36%	284	1.22%
*Citrobacter spp.*	17	0.56%	223	1.10%	240	1.03%
*Acinetobacter baumannii*	25	0.82%	125	0.62%	150	0.64%
*Proteus spp.*	16	0.53%	103	0.51%	119	0.51%
*Enterobacter spp.*	3	0.10%	43	0.21%	46	0.20%
*Klebsiella spp.*	6	0.20%	33	0.16%	39	0.17%
*Serratia spp.*	6	0.20%	29	0.14%	35	0.15%

*cUTI* complicated urinary tract infection, *TR* triple resistant

^a^Adds up to > 100% due to polymicrobial infections

**Table 2:** Baseline characteristics

	TR	%	non-TR	%	***P***-value
	*N* = 3040 (13.03%)		*N* = 20,291 (86.97%)		
Mean age, years (SD)	66.9 (16.6)		65.1 (18.0)		< 0.001
Gender: male	1752	57.63%	9685	47.73%	< 0.001
Race					
White	2186	71.91%	15,482	76.30%	< 0.001
Black	543	17.86%	2759	13.60%	
Other	282	9.28%	1919	9.46%	
Unknown	29	0.95%	131	0.65%	
Hispanic Ethnicity	164	5.39%	1087	5.36%	0.931
Admission Source					
Non-healthcare facility (including from home)	2307	75.89%	16,518	81.41%	< 0.001
Clinic	201	6.61%	1299	6.40%	
Transfer from SNF, ICF	324	10.66%	1041	5.13%	
Transfer from another non-acute care facility	122	4.01%	779	3.84%	
Other	86	2.83%	654	3.22%	
Admission type					
Medical	2724	89.61%	17,890	88.17%	0.021
Surgical	316	10.39%	2401	11.83%	
Elixhauser Comorbidities					
Congestive heart failure	705	23.19%	3999	19.71%	< 0.001
Valvular disease	166	5.46%	1339	6.60%	0.017
Pulmonary circulation disease	133	4.38%	886	4.37%	0.983
Peripheral vascular disease	265	8.72%	1574	7.76%	0.067
Paralysis	763	25.10%	2671	13.16%	< 0.001
Other neurological disorders	876	28.82%	4246	20.93%	< 0.001
Chronic pulmonary disease	798	26.25%	4568	22.51%	< 0.001
Diabetes without chronic complications	852	28.03%	4664	22.99%	< 0.001
Diabetes with chronic complications	490	16.12%	2800	13.80%	0.001
Hypothyroidism	527	17.34%	3237	15.95%	0.053
Renal failure	959	31.55%	5683	28.01%	< 0.001
Liver disease	114	3.75%	866	4.27%	0.184
Peptic ulcer disease with bleeding	8	0.26%	54	0.27%	0.976
AIDS	10	0.33%	50	0.25%	0.402
Lymphoma	22	0.72%	212	1.04%	0.098
Metastatic cancer	91	2.99%	753	3.71%	0.048
Solid tumor without metastasis	121	3.98%	1016	5.01%	0.014
Rheumatoid arthritis/collagen vascular	104	3.42%	934	4.60%	0.003
Coagulopathy	271	8.91%	1916	9.44%	0.351
Obesity	619	20.36%	3576	17.62%	< 0.001
Weight loss	476	15.66%	2393	11.79%	< 0.001
Fluid and electrolyte disorders	1695	55.76%	10,940	53.92%	0.057
Chronic blood loss anemia	53	1.74%	306	1.51%	0.326
Deficiency anemia	833	27.40%	4870	24.00%	< 0.001
Alcohol abuse	46	1.51%	450	2.22%	0.012
Drug abuse	88	2.89%	655	3.23%	0.329
Psychosis	139	4.57%	801	3.95%	0.102
Depression	635	20.89%	3454	17.02%	< 0.001
Hypertension	2063	67.86%	13,378	65.93%	0.036
Charlson Comoribidity Score					
0	344	11.32%	4046	19.94%	< 0.001
1	413	13.59%	3534	17.42%	
2	669	22.01%	3893	19.19%	
3	517	17.01%	3052	15.04%	
4	375	12.34%	2069	10.20%	
5+	722	23.75%	3697	18.22%	
Mean (SD)	3.1 (2.3)		2.6 (2.4)		< 0.001
Median [IQR]	3 [2,4]		2 [1,4]		< 0.001
Hospital Characteristics					
Census region					
Midwest	956	31.45%	6829	33.66%	< 0.001
Northeast	370	12.17%	2653	13.07%	
South	1406	46.25%	8412	41.46%	
West	308	10.13%	2397	11.81%	
Number of Beds					
< 100	121	3.98%	1010	4.98%	
100 to 199	373	12.27%	2623	12.93%	0.001
200 to 299	667	21.94%	4138	20.39%	
300 to 399	524	17.24%	3051	15.04%	
400 to 499	504	16.58%	3567	17.58%	
500+	851	27.99%	5902	29.09%	
Teaching	1258	41.38%	8482	41.80%	0.661
Urban	2623	86.28%	17,599	86.73%	0.815
C3R Rate at Hospital Level					
Mean (SD)	17.0% (7.6%)		14.8% (6.8%)		< 0.001
Median [IQR]	16.3% [12.4, 20.9%]		14.4% [10.2, 17.7%]		< 0.001
TR Rate at Hospital Level					
Mean (SD)	15.7% (6.4%)		12.6% (5.7%)		< 0.001
Median [IQR]	15.1% [11.3, 18.8%]		12.2% [9.4, 16.1%]		< 0.001

*MDR* multidrug resistant, *SD* standard deviation, *SNF* skilled nursing facility, *ICF* intermediate care facility, *AIDS* acquired immune deficiency syndrome, *IQR* interquartile range, *C3R* 3rd generation cephalosporin-resistant, *CR* carbapenem resistant

TR was associated with increase in some illness severity markers relative to non-TR (need for MV and ICU), though others were similar between the two groups (Table 3). Though the majority of all infections were monomicrobial (71.8% TR vs. 84.4% non-TR), patients with TR were more likely to have a polymicrobial cUTI. Similarly, while > 95% of all cUTI was CO, HCA cUTI was more common in TR patients than among non-TR (49.5% vs. 36.2%, *p* < 0.001). Consequently, more patients with TR had experienced a hospitalization and antimicrobial treatment within 90 days prior to the index hospitalization, and had within the same time period an isolate exhibiting resistance to one of the antibiotics or classes (Table 3). Despite more frequent use of such broad-spectrum empiric coverage as piperacillin-tazobactam and carbapenems, the group with TR, compared to non-TR, were more likely to receive IET (19.6% vs. 5.4%, *p* < 0.001).

**Table 3:** Infection and treatment characteristics

	TR	%	non-TR	%	*P*-value
	*N* = 3040		*N* = 20,291		
**Infection**					
Illness severity measures by day 2 from onset					
ICU admission	677	22.27%	3774	18.60%	< 0.001
Mechanical ventilation	213	7.01%	1012	4.99%	< 0.001
Vasopressors	183	6.02%	1333	6.57%	0.252
Dialysis	68	2.24%	389	1.92%	0.235
Severe sepsis	537	17.66%	3549	17.49%	0.814
Severe sepsis POA	523	17.20%	3382	16.67%	0.460
Septic shock	386	12.70%	2274	11.21%	0.016
Septic shock POA	360	11.84%	2049	10.10%	0.003
Monomicrobial	2184	71.84%	17,115	84.35%	< 0.001
Polymicrobial					
2 organisms	758	24.93%	2837	13.98%	< 0.001
3 or more organisms	98	3.22%	339	1.67%	
Infection characteristics					
Community-onset	2970	97.70%	19,472	95.96%	< 0.001
Community-acquired	1465	48.19%	12,134	59.80%	
Healthcare-associated	1505	49.51%	7338	36.16%	
Hospital-onset	70	2.30%	819	4.04%	< 0.001
Type of cUTI					
CAUTI	747	24.57%	8984	44.28%	
non-CAUTI-cUTI	2293	75.43%	11,307	55.72%	< 0.001
Culture source					
Blood only	12	0.39%	138	0.68%	
Urine only	917	30.16%	6321	31.15%	0.090
Blood and urine	2111	69.44%	13,832	68.17%	
Time to cUTI					
Mean (SD)	1.3 (2.3)		1.5 (3.0)		0.002
Median [IQR]	1 [1,1]		1 [1,1]		< 0.001
Prior hospitalization within 90 days	1250	41.12%	6074	29.93%	< 0.001
Antibiotics within 90 days prior to admission	1101	36.22%	4733	23.33%	< 0.001
Antibiotics during index hospitalization prior to cUTI Index Day	225	7.40%	1594	7.86%	0.384
C3-R organism within 90 days prior to admission	356	11.71%	386	1.90%	< 0.001
FQ-R organism within 90 days prior to admission	588	19.34%	1436	7.08%	< 0.001
TMP/SMZ-R organism within 90 days prior to admission	495	16.28%	799	3.94%	< 0.001
FFM-R organism within 90 days prior to admission	1	0.03%	1	0.00%	0.244
NFT-R organism within 90 days prior to admission	307	10.10%	625	3.08%	< 0.001
TR organism within 90 days prior to admission	411	13.52%	327	1.61%	< 0.001
**Treatment**					
Antibiotics administered by day 2 from onset					
Antipseudomonal penicillins with beta-lactamase inhibitor	1107	36.41%	6507	32.07%	< 0.001
Extended spectrum cephalosporins	1651	54.31%	13,576	66.91%	< 0.001
Antipseudomonal floroquinolones	761	25.03%	6672	32.88%	< 0.001
Aminoglycosides	295	9.70%	1722	8.49%	0.025
Non-PIP/TAZ penicillins with beta-lactamase inhibitors	37	1.22%	386	1.90%	0.008
PIP/TAZ	1107	36.41%	6505	32.06%	< 0.001
Tetracyclines	35	1.15%	238	1.17%	0.918
Folate pathway inhibitors	39	1.28%	390	1.92%	0.014
Polymyxins	6	0.20%	22	0.11%	0.187
Antipseudomonal cephalosporins	646	21.25%	3913	19.28%	0.011
Carbapenems	1317	43.32%	3287	16.20%	< 0.001
Aztreonam	107	3.52%	784	3.86%	0.356
Tygecycline	31	1.02%	61	0.30%	< 0.001
C3	1245	40.95%	11,440	56.38%	< 0.001
FQ	768	25.26%	6704	33.04%	< 0.001
TMP/SMZ	39	1.28%	390	1.92%	0.014
FFM	6	0.20%	42	0.21%	0.913
NFT	38	1.25%	233	1.15%	0.625
Empiric treatment appropriateness					
Non-IET	2124	69.87%	15,990	78.80%	< 0.001
Inapproprite Empiric Treatment (IET)	597	19.64%	1101	5.43%	
Indeterminate	319	10.49%	3200	15.77%	

*MDR* multidrug resistant, *SD* standard deviation, *IQR* interquartile range, *ICU* intensive care unit, *POA* present on admission, *cUTI* complicated urinary tract infection, *CAUTI* catheter-associated UTI, *R* resistant, *C3* 3rd generation cephalosporin, *FQ* fluoroquinolone, *TMP/SMZ* trimethoprim-sulfamethoxazole, *FFM* fofsomycin, *NFT* nitrofurantoin, *R* resistant, *PIP/TAZ* piperacillin-tazobactam, *IET* inappropriate empiric therapy

The unadjusted outcomes are depicted in Supplemental Table 3. As for adjusted outcomes, although TR was not associated with a rise in hospital mortality or 30-day readmission rate, it was associated with greater hospital LOS and costs (Table 4). Testing for interactions revealed that TR affects both LOS and costs differently among patients with CAUTI versus non-CAUTI cUTI. Namely, though the entire cUTI cohort’s mean TR-attributable cost excess was $754 (95% CI $406, $1103, *p* < 0.001), it was $125 (95% CI -$275, $525, *p* = 0.540) for CAUTI and $1637 (95% CI $1045, $2229, *p* < 0.001) for non-CAUTI cUTI (Supplemental Fig. 1). Similarly, though non-CAUTI-cUTI patients had a TR-attributable increase in post-infection onset LOS of 0.62 days (95% CI 0.35, 0.88, p < 0.001), CAUTI patients stayed longer regardless of their TR status, driving the overall cohort’s LOS excess related to TR to 0.34 days (95% CI 0.18, 0.49, *p* < 0.001). The results were similar for the total LOS (data not shown).

**Table 4:** Adjusted contribution of triple resistance to outcomes

Outcome	Metric	Point estimate	95% confidence interval	***P*** value
Mortality	Odds ratio	1.03	(0.78, 1.35)	0.844
30-day readmission	Odds ratio	1.04	(0.94, 1.16)	0.429
Hospital cost	Excess $	$754	($406, $1103)	< 0.001
Total LOS	Excess days	0.28 days	(0.12, 0.44)	< 0.001
Post-infection onset LOS	Excess days	0.34 days	(0.18, 0.49)	< 0.001
CDI^a^	Odds ratio	1.49	(0.95, 2.32)	0.08
cUTI relapse	Odds ratio	0.82	(0.44, 1.54)	0.535

*LOS* length of stay, *CDI C. difficile* infection, *cUTI* complicated urinary tract infection

^a^Incident CDI *n* = 118 (0.5%)

Examining the impact of IET on the outcomes of cUTI revealed similar interaction with the type of cUTI in the mortality estimate. That is, in patients with non-CAUTI cUTI, IET raised the risk of mortality (OR 2.44; 95% CI 1.30, 4.56, *p* = 0.005), while this effect was absent in the CAUTI group (1.26; 95% CI 0.77, 2.04, *p* = 0.355). Additionally, IET was associated with increases in marginal hospital costs ($1364 in total costs; 95% CI $923, $1805, *p* < 0.001), overall LOS (0.66 days; 95% CI 0.46, 0.86, *p* < 0.001), and post-infection LOS (0.73 days; 95% CI 0.52, 0.94, p < 0.001) in the cUTI cohort overall.

Although incident CDI occurred in only 0.5% of the cohort, TR increased the risk of its occurrence, but did not reach statistical significance (OR 1.49, 95% CI 0.95, 2.32, *p* = 0.08, Table 4). TR was not associated with an increased risk of cUTI recurrence. Notably, the development of TR was rare in the non-TR group (0.45%, Supplemental Table 3).

## Discussion

In this large multicenter retrospective analysis of US hospitals TR is present in approximately 13% of patients with a cUTI. That is, nearly 1 in 8 patients with a cUTI, the vast majority of which are community-onset, are infected with a pathogen that is resistant to at least 3 of the following antimicrobials/classes: 3rd generation cephalosporins, fluoroquinolones, trimethoprim-sulfamethoxazole, fosfomycin, and nitrofurantoin. Not surprisingly, the presence of a TR pathogen increases the risk for IET. Interestingly, both TR and IET impact the outcomes differentially, depending on the type of cUTI. TR and IET are not significantly associated with higher costs or LOS in CAUTI, which are already high in this subset of cUTI. In contrast, non-CAUTI cUTI incur both higher costs and LOS with TR and IET, and higher mortality with IET. Indeed, IET results in an increase in the LOS of nearly 1 day, and excess costs of over $1300. TR and IET were important drivers in non-CAUTI cUTI of these outcomes even in the face of overall high severity of illness, with nearly ¼ requiring ICU, and over 10% with septic shock. Finally, though its overall incidence was low, CDI was associated more frequently with TR than non-TR, though this difference failed to reach statistical significance likely due to its low incidence.

We have specifically avoided the language of “multidrug resistant” (MDR) in our analysis. The US Centers for Disease Control and Prevention defines as MDR an isolate that is resistant to at least one antibiotic in three or more drug classes [14]. If those classes are not routinely employed as treatment for a specific syndrome, however, the term has little practical application to front line physicians while they make treatment choices. Hence, instead of enumerating the frequency of in vitro non-susceptibility to choices one might never consider in cUTI, our study addressed agents regularly given in this specific setting, so as to identify the characteristics of patients in whom the “standard” regimens might fail.

In contrast to some other populations, where both resistance and IET contribute to a rise in mortality we did not find that to be the case in the overall cUTI cohort [15–18]. This is likely due to the low (2%) baseline mortality rate compared to infections such as healthcare- and ventilator-associated pneumonia and sepsis, where crude case fatality ranges from 10 to 40% [11, 12, 15–22]. This lack of a mortality effect in our study mirrors that in other low-risk of death infections, including severe skin infection [23]. However, we identified a differential effect for both TR and IET on the outcomes, depending on the type of cUTI – CAUTI vs. non-CAUTI cUTI. Namely, while TR and IET alter the outcomes in the latter, they do not appear to have an impact in the former, suggesting that these two groups should be analyzed separately vis-à-vis their treatment outcomes.

The relationship between TR and IET and excess costs in our population is also consistent with the findings of others. Work in multiple other infections, including lower mortality syndromes, regularly illustrates that resistance contributes to IET, and delayed or inappropriate empiric therapy increases the LOS and, in turn, adds to costs [23]. Our findings specific to cUTI are novel and add to the present literature. Importantly, although an absolute increase in LOS of less than one day may appear trivial at first glance, our estimate of IET-attributable excess of 0.7 days represents approximately 10% of the entire LOS for the average patient with a cUTI. Similarly, even though TR- and IET-attributable excess costs of $754 and $1364, respectively, may seem modest, from a hospital perspective these costs can quickly become substantial, given the combination of frequency of admissions with a cUTI and the already strained reimbursement rates.

One additional novel aspect of our study is that we quantified incident CDI in cUTI. Although the overall rate was lower than in other hospitalized populations, TR did increase the risk of this infection [13, 24–26]. Though we did not specifically examine the implications of CDI on 30-day readmission, it is likely another potential source of LOS and cost rise.

What are the practical implications of our observations? First, certain exposures remain associated with TR. Some of these relate to prior interaction with the healthcare system and suggest that physicians must strive to determine a patient’s prior healthcare and antibiotic exposures when making prescribing decisions. Though the concept of “healthcare-associated infection” may currently be out of favor in select formal guidelines, the evidence indicates that not all community-onset infections pose the same risk for resistance and the accompanying concern for IET [27]. Second, pre-test probability matters. That is, readers should not interpret our findings as a call to abandon current practices and move to selecting broader-acting antimicrobials for first line therapy in cUTI; that would only serve to foster more resistance. Rather, our observations stress the imperative for physicians to have granular data on local microbiology as a function of the syndromes they treat.

Our study has a number of limitations and strengths. The observational nature of the study predisposes it to multiple threats to validity, particularly a selection bias. By defining the cohort prospectively, we attempted to mitigate the magnitude of this bias. Misclassification is an issue, particularly when using administrative data. To deal with this, we used a previously published, though not clinically validated, algorithm, excluded other potential sources of infection, and included microbiology specimens, pharmacy data, and dates of cultures and treatments to minimize its magnitude. If present, however, this type of misclassification would drive the differences between groups toward null. At the same time, we could not differentiate between infection and colonization. Confounding is a potential problem in all observational studies. We performed multivariable modeling to minimize its impact using many confounders. However, some residual confounding may remain. Because this is a large multicenter geographically representative database, generalizability is of minimal concern, though we must caution that our results apply only to hospitalized patients, and not those treated in the community. Despite these limitations, this is the largest multicenter cohort study to examine the prevalence, time trends and outcomes of antimicrobial resistance in cUTI.

## Conclusions

In summary, we have demonstrated that resistance to combinations of regularly used antimicrobials is prevalent and on the rise in the most common cUTI organisms in the US hospitals. Though increasing resistance alone does not impact hospital mortality, it does expose patients to an elevated risk of worsened outcomes through increasing the likelihood of inappropriate empiric therapy.

## Supplementary Information

**Additional file 1.**

## Data Availability

The data that support the findings of this study are available from Premier Research but restrictions apply to the availability of these data, which were used under license for the current study, and so are not publicly available. Data are however available from the authors upon reasonable request and with permission of Premier Research.

## Abbreviations

cUTI: Complicated urinary tract infection
CAUTI: Catheter-associated urinary tract infection
CO: Community-onset
HO: Hospital-onset
ESBL: Extended-spectrum beta-lactamase
IET: Inappropriate empiric therapy
HCA: Healthcare-associated
CA: Community-acquired
R: Resistant
C3: 3rd generation cephalosporins
FQ: Fluoroquinolones
TMP-SMZ: Trimethoprim-sulfamethoxazole
FFM: Fosfomycin
NTF: Nitrofurantoin
TR: Triple-drug resistant
LOS: Length of stay
CDI: *C. difficile* infection
IQR: Interquartile range
CI: Confidence interval
MDR: Multidrug resistant
